# Supplementary material for: Small‐molecule inhibition of aging‐associated chromosomal instability delays cellular senescence
Source: EMBO Rep. 2020 Mar 5;21(5):e49248. doi: 10.15252/embr.201949248 (PMC7202060; doi:10.15252/embr.201949248)
Supplement: Supplementary file 1 — Appendix [file EMBR-21-e49248-s001.pdf]

Appendix for Barroso-Vilares et al., **Small molecule inhibition of aging-associated chromosomal instability delays cellular senescence (2020)**

Table of Contents

1. Appendix Figure S1 2
2. Appendix Figure S2 3
3. Appendix Figure S3 4
4. Appendix Table S1 5

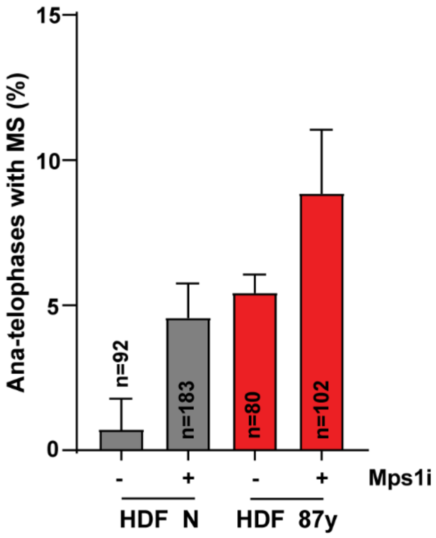

Appendix Figure S1 (related to Fig 1). Increased frequency of chromosome mis-segregation in elderly vs. neonatal cells following Mps1 partial inhibition.

Percentage of ana-telophases with chromosome mis-segregation (MS) in neonatal vs. elderly cells treated with DMSO (-) or 500 nM AZ3146/Mps1i (+) for 4 hours as scored by Fluorescence *in situ* hybridization (FISH) using centromeric probes against chromosomes 7, 12 and 18.

Data information: values shown are mean ± s.d. of at least two independent experiments.

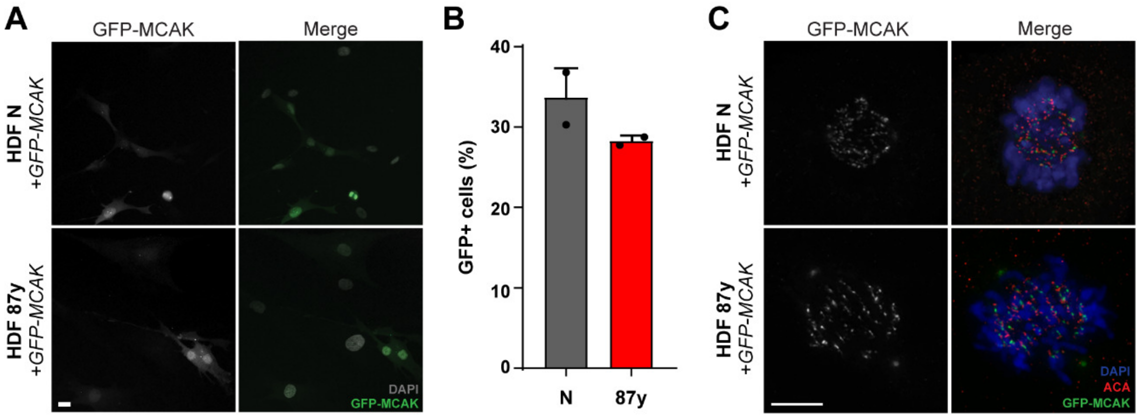

Appendix Figure S2 (related to Fig 2). GFP-MCAK overexpression in young and elderly cells.

(**A, B**) Representative images (A) and percentages (B) of GFP-MCAK positive cells in total cell populations of neonatal (HDF N) or elderly (HDF 87y) after lentiviral transduction. Scale bar, 20µm.

(**C**) Representative images of mitotic cells overexpressing GFP-MCAK in neonatal and elderly cell populations transduced with lentiviruses. Scale bar, 5µm.

Data Information: Values shown in (B) are mean ± s.d. of two independent experiments, which are indicated as dots.

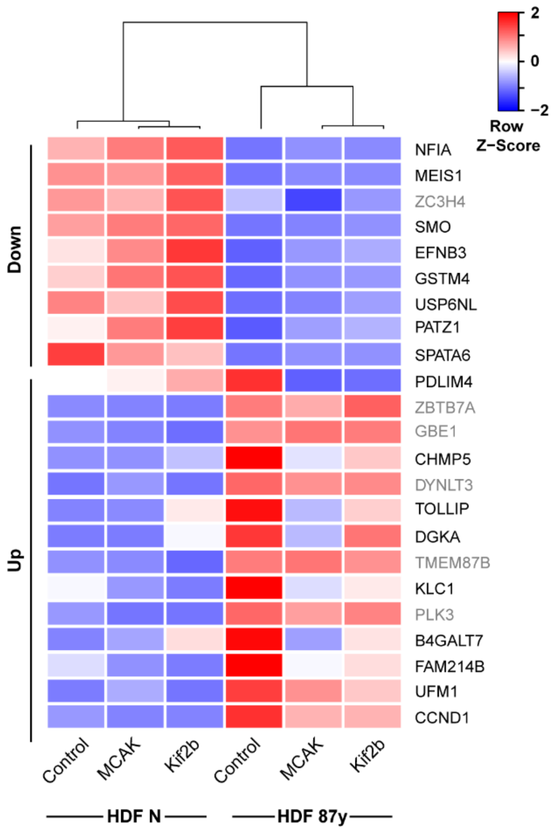

Appendix Figure S3 (related to Fig 3). Kinesin-13 overexpression rescues senescence core gene expression signature in elderly fibroblasts.

Heatmap of differentially expressed senescence core signature genes (see Dataset EV3). Down and Up refer to the expected changes reported for senescent cells. Gene symbols highlighted in grey indicate genes whose expression remained unaltered following Kinesin-13 overexpression. Z-score row color intensities indicate higher (red) to lower (blue) expression.

Appendix Table S1. List of primers used for lenviral plasmid assembly and quantitative PCR.

Gene/Fragment name	Gene Symbol	Forward primer (5' to 3')	Reverse primer (5' to 3')	Experiment
BamHI-GFP-MCAK-NotI	-	TATCGCGGATCCATGGTGAGCA AGGGCGAGGAG	TATATAGCGGCCGCTCACTGGGGC CGTTTCTTGCTG	LP
NotI-GFP-Kif2b-MluI	-	TATATAGCGGCCGCATGGTGAG CAAGGGCGAGGAG	TATACGACGCGTTCACTCTACCTT GCTCTTCA	LP
BamHI-EOS-α-tubulin-NotI	-	CGCCGCGGATCCATGAGTGCGA TTAAGCCAGAC	TATATAGCGGCCGCTTAGTATTCC TCTCCTTCTTCC	LP
Aurora kinase B	*AURKB*	CGCAGAGAGATCGAAATCCAG	AGATCCTCCTCCGGTCATAAAA	qPCR
Beta-1,4-Galactosyltransferase 7	*B4GALT7*	CACTACCGGCTGTGCAATG	ACCCAGTTGTGATTCCCGAG	qPCR
Cyclin D1	*CCND1*	AGCTGTGCATCTACACCGAC	GAAATCGTGCGGGGTCATTG	qPCR
Cyclin-Dependent Kinase Inhibitor 1A	*CDKN1A/P21*	TGGACCTGGAGACTCTCAGG	CGGATTAGGGCTTCCTCTTGG	qPCR
Cyclic GMP-AMP synthase	*CGAS*	ACGTGCTGTGAAAACAAAGAAG	GTCCCACTGACTGTCTTGAGG	qPCR
Family With Sequence Similarity 214 Member B	*FAM214B*	CACCATCCAAGTGACCTTATTTA ACC	AGAAGTCAAAGGTCACAAGGAAC AT	qPCR
Hypoxanthine phosphoribosyltransferase 1	*HPRT1*	TGACCAGTCAACAGGGGACA	CTGCATTGTTTTGCCAGTGTCAA	qPCR
kinesin family member 2C	*KIF2C/MCAK*	CTCAGTTCGGAGGAAATCATGTC	TGCTCTTCGATAGAATCAGTCAC	qPCR
NDC80 kinetochore complex component	*NDC80/HEC1*	CCTCTCCATGCAGGAGTTAAGA	GGTCTCGGGTCCTTGATTTTCT	qPCR
Polo like kinase 1	*PLK1*	AAAGAGATCCCGGAGGTCCTA	GGCTGCGGTGAATGGATATTTC	qPCR
TATA-box binding protein	*TBP*	GAGCCAAGAGTGAAGAACAGTC	GCTCCCCACCATATTCTGAATCT	qPCR
Toll Interacting Protein	*TOLLIP*	GACTTCCTCCGCATCACGC	CCAACTTTGCCTGTACCACC	qPCR

LP - lentiviral plasmid assembly; qPCR - quantitative PCR
