# Supplementary material for: Small‐molecule inhibition of aging‐associated chromosomal instability delays cellular senescence
Source: EMBO Rep. 2020 Mar 5;21(5):e49248. doi: 10.15252/embr.201949248 (PMC7202060; doi:10.15252/embr.201949248)
Supplement: Supplementary file 2 — Expanded View Figures PDF [file EMBR-21-e49248-s002.pdf]

Expanded View Figures

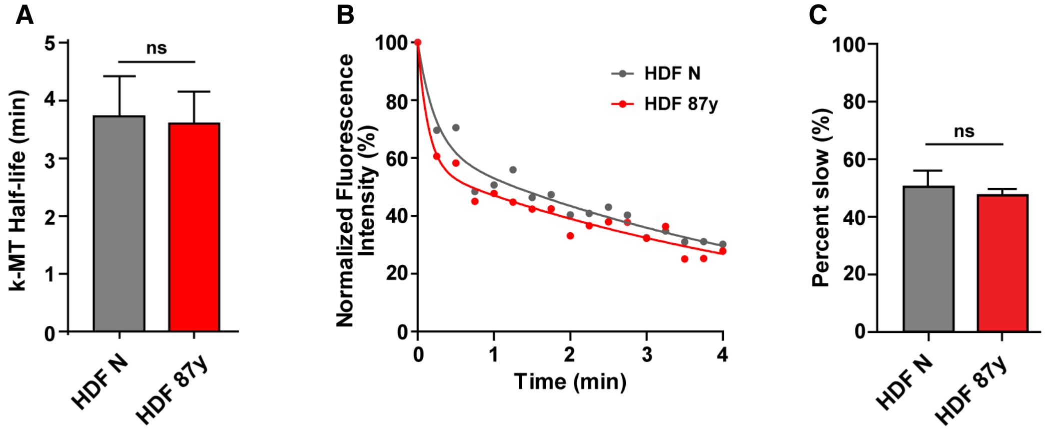

Figure EV1. Similar k-MT detachment rates in young and elderly fibroblasts (related to Fig 1).

A Average k-MT half-life for $n = 12$ neonatal (HDF N) and elderly (HDF 87 years) metaphase cells expressing mEOS-Tubulin.

B Examples of normalized fluorescence dissipation after photoconversion in neonatal versus 87 years metaphase cells that are representative of the average k-MT half-life shown in (A).

C Percentage of photoconverted fluorescence intensity attributable to the slow decay process for $n = 12$ neonatal versus 87 years cells.

Data Information: Values shown are mean ± SEM of at least two independent experiments. ns $P > 0.05$ by two-tailed *t*-test.

Figure EV2. Decreased levels of main regulators of k-MT dynamics in mitotic cells from elderly donors (related to Fig 1).

A Relative *AURKB*, *PLK1*, *HEC1*, and *MCAK* transcript levels in total RNA of mitotic fibroblasts from elderly (HDF 77/83/87 years; $n > 5$ replicates) versus neonatal (HDF N/N; $n \geq 3$ replicates) donors. *TBP* and *HPRT1* were used as reference genes.

B Western blot analysis (left) and quantification (right) of Aurora B, Plk1, Hec1, and MCAK protein levels in mitotic extracts of elderly (HDF 85/87 years; $n \geq 3$ replicates per age) normalized against neonatal (HDF N/N; $n = 5$ replicates per neonatal sample) fibroblasts. GAPDH was used as loading control.

C–J Representative images and immunofluorescence intensity levels of (C, G) Aurora B, (D, H) Plk1, (E, I) Hec1, and (F, J) MCAK, in $n = 100$ kinetochores of mitotic cells (N versus 87 years). Intensity levels were normalized to ACA centromere staining. Scale bars, 5 µm.

Data Information: All values shown are mean ± SD of at least two independent experiments. ns $P > 0.05$, *$P < 0.05$, **$P < 0.01$, ***$P < 0.001$, ****$P < 0.0001$ in comparison to neonatal by two-tailed Mann–Whitney test.
Source data are available online for this figure.

 © 2020 The Authors

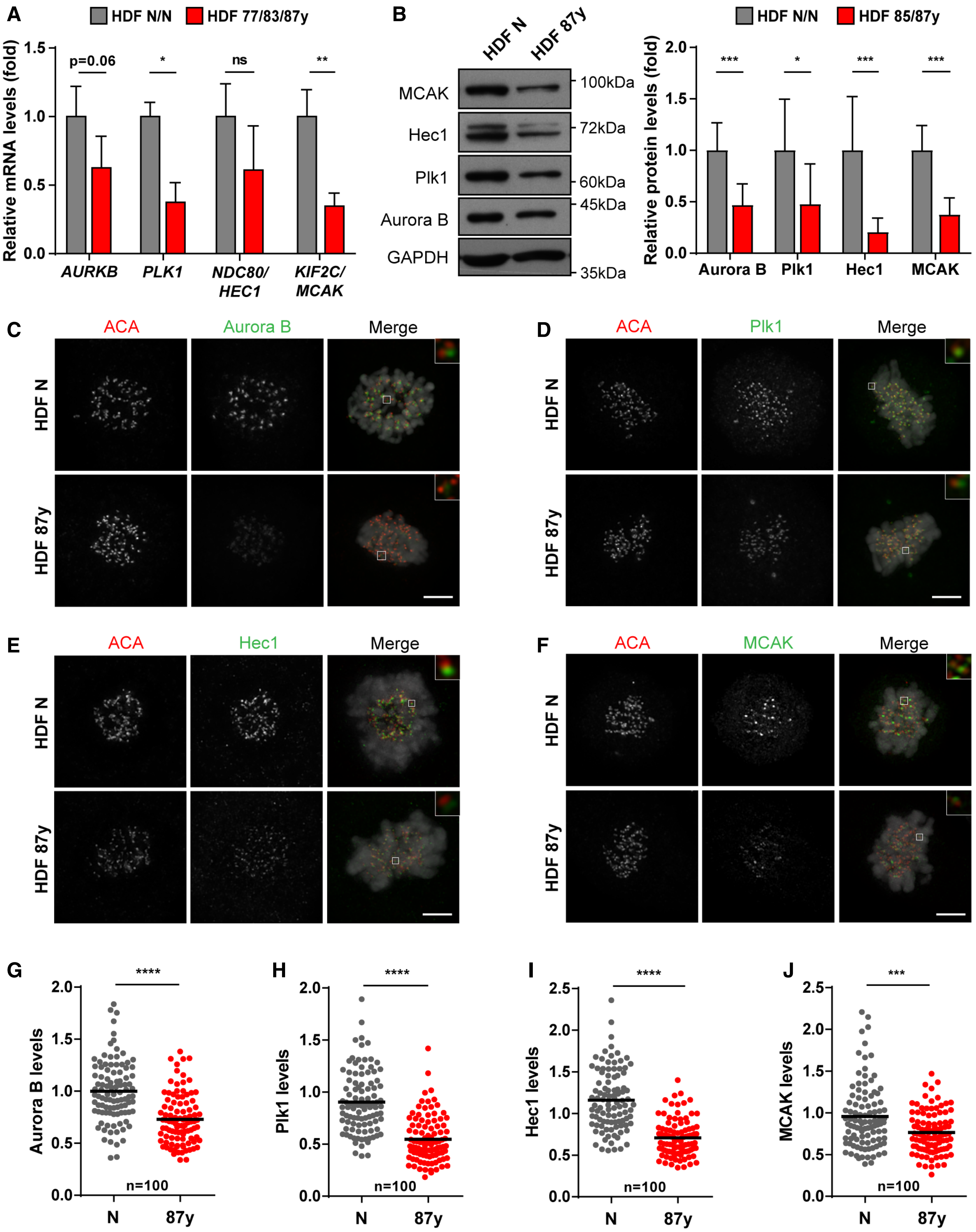
A
HDF N/N
HDF 77/83/87y
Relative mRNA levels (fold)
p=0.06
*
ns
**
AURKB
PLK1
NDC80/HEC1
KIF2C/MCAK
B
HDF N
HDF 87y
MCAK
Hec1
Plk1
Aurora B
GAPDH
100kDa
72kDa
60kDa
45kDa
35kDa
HDF N/N
HDF 85/87y
Relative protein levels (fold)
***
*
***
***
Aurora B
Plk1
Hec1
MCAK
C
ACA
Aurora B
Merge
HDF N
HDF 87y
D
ACA
Plk1
Merge
HDF N
HDF 87y
E
ACA
Hec1
Merge
HDF N
HDF 87y
F
ACA
MCAK
Merge
HDF N
HDF 87y
G
Aurora B levels
****
n=100
N
87y
H
Plk1 levels
****
n=100
N
87y
I
Hec1 levels
****
n=100
N
87y
J
MCAK levels
***
n=100
N
87y

Figure EV2.

Figure EV3. cGAS engagement in aging-associated micronucleation and senescence phenotypes (related to Fig 3).

A Relative cytokine levels (fold change) in cell medium supernatants of elderly (HDF 87 years) cells normalized to neonatal (HDF N) cells (n = 2 replicates per age, shown as individual datapoints).

B Relative *CGAS* transcript levels in total RNA of fibroblasts from elderly (HDF 87 years; n = 5 replicates) versus neonatal (HDF N; n = 6 replicates) donors. *TBP* and *HPRT1* were used as reference genes.

C, D Representative images (C) and quantification (D) of intact (cGAS$^-$/Rb$^+$) or disrupted (cGAS$^+$/Rb$^-$) micronuclei (MN) in $n \geq$ 2,958 immunostained cells. Arrowheads indicate micronuclei. Scale bar, 10 μm.

E Western blot analysis of cGAS protein levels following siRNA depletion (sicGAS). GAPDH is shown as loading control.

F Quantification of cGAS-positive micronuclei in n = cells following sicGAS depletion.

G Percentage of n = cells staining positive for double immunostaining of Cdkn1a/p21 (cell cycle inhibitor) and 53BP1 ($\geq$ 1 foci; DNA damage) senescence biomarkers following cGAS depletion.

H Percentage of n = cells staining positive for SA-β-galactosidase (SA-β-gal) activity after cGAS depletion.

I Percentage of cGAS-positive micronuclei in $n \geq$ 1,674 cells of neonatal and elderly cells transduced with empty (control), GFP-MCAK, or GFP-Kif2b lentiviral plasmids.

Data Information: All values shown are mean ± SD of at least two independent experiments. ns $P > 0.05$, *$P < 0.05$, **$P < 0.01$, ***$P < 0.001$, ****$P < 0.0001$ in comparison to neonatal by two-tailed (D, F–I) Mann–Whitney and (B) chi-square tests.
Source data are available online for this figure.

 © 2020 The Authors

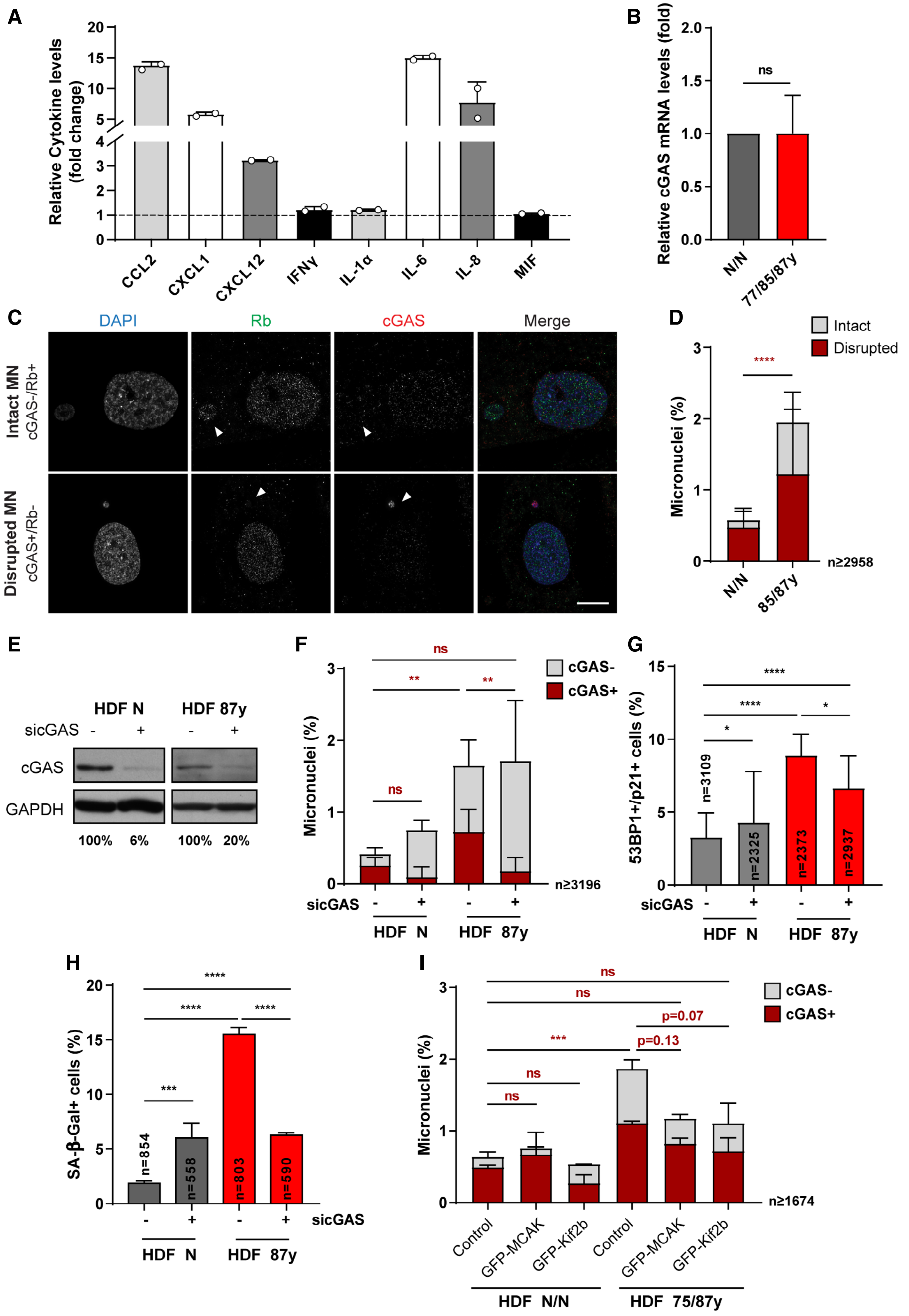
A
Relative Cytokine levels (fold change)
CCL2
CXCL1
CXCL12
IFNγ
IL-1α
IL-6
IL-8
MIF
B
Relative cGAS mRNA levels (fold)
ns
N/N
77/85/87y
C
DAPI
Rb
cGAS
Merge
Intact MN cGAS-/Rb+
Disrupted MN cGAS+/Rb-
D
Intact
Disrupted
Micronuclei (%)
****
N/N
85/87y
n≥2958
E
HDF N
HDF 87y
sicGAS - + - +
cGAS
GAPDH
100% 6% 100% 20%
F
Micronuclei (%)
ns
**
**
ns
cGAS-
cGAS+
n≥3196
sicGAS - + - +
HDF N
HDF 87y
G
53BP1+/p21+ cells (%)
****
****
*
*
n=3109
n=2325
n=2373
n=2937
sicGAS - + - +
HDF N
HDF 87y
H
SA-β-Gal+ cells (%)
****
****
****
***
n=854
n=558
n=803
n=590
- + - + sicGAS
HDF N
HDF 87y
I
Micronuclei (%)
ns
ns
p=0.07
***
p=0.13
ns
ns
cGAS-
cGAS+
n≥1674
Control
GFP-MCAK
GFP-Kif2b
Control
GFP-MCAK
GFP-Kif2b
HDF N/N
HDF 75/87y

Figure EV3.

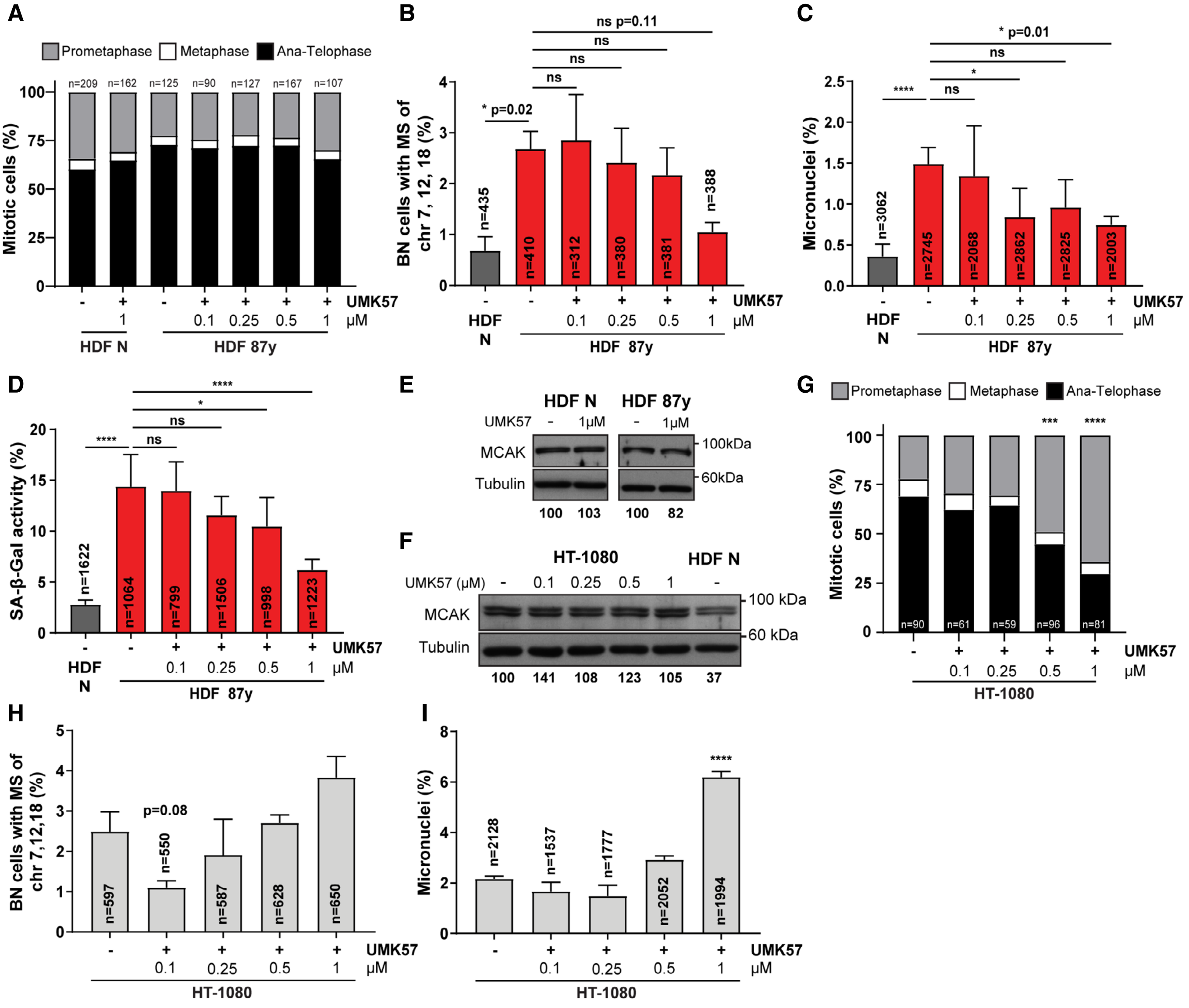

Figure EV4. UMK57 optimal concentration for suppressing CIN in dermal fibroblasts versus HT-1080 cancer cells (related to Fig 4).

A Percentage of n = mitotic cells of neonatal (N) and elderly (87 years) human dermal fibroblast (HDF) cultures in prometaphase, metaphase, or ana-telophase upon 24 h of exposure to DMSO (–) or increasing concentrations of UMK57 (+) as indicated.

B Cytochalasin D-induced binucleated (BN) cells with mis-segregation (MS) of chromosomes 7, 12, and 18 in neonatal (HDF N) and elderly samples (HDF 87 years) after 24-h treatment with DMSO (–) or increasing concentrations of UMK57 (+) as indicated.

C Percentage of micronuclei in neonatal (N) and elderly (87 years) n = cells treated with DMSO (–) or increasing concentrations of UMK57 (+) as indicated for 24 h.

D Percentage of neonatal versus 87 years n = cells staining positive for SA-β-gal activity upon 24-h treatment with DMSO (–) or increasing concentrations of UMK57 (+) as indicated.

E Western blot analysis of MCAK protein levels in mitotic extracts of neonatal (HDF N) and elderly (HDF 87 years) fibroblasts following treatment with DMSO (–) or 1 μM UMK57 (+). Tubulin is shown as loading control.

F Western blot analysis of MCAK protein levels in mitotic extracts of HT-1080 cells or neonatal fibroblasts treated with DMSO (–) or increasing concentrations of UMK57 (+) for 24 h, as indicated. Tubulin is shown as loading control.

G Percentage of n = HT-1080 mitotic cells in prometaphase, metaphase, or ana-telophase upon 24-h treatment with DMSO (–) or increasing concentrations of UMK57 (+) as indicated.

H Percentage of binucleated (BN) HT-1080 cells with mis-segregation (MS) of chromosomes 7, 12, and 18 upon 24-h treatment with DMSO (–) or increasing concentrations of UMK57 (+) as indicated.

I Percentage of micronuclei in n = HT-1080 cells following 24-h treatment with DMSO (–) or increasing concentrations of UMK57 (+) as indicated.

Data information: All values shown are mean ± SD of at least two independent experiments. ns $P > 0.05$, *$P < 0.05$, ***$P < 0.001$, ****$P < 0.0001$ by two-tailed chi-square test.

Source data are available online for this figure.

 © 2020 The Authors

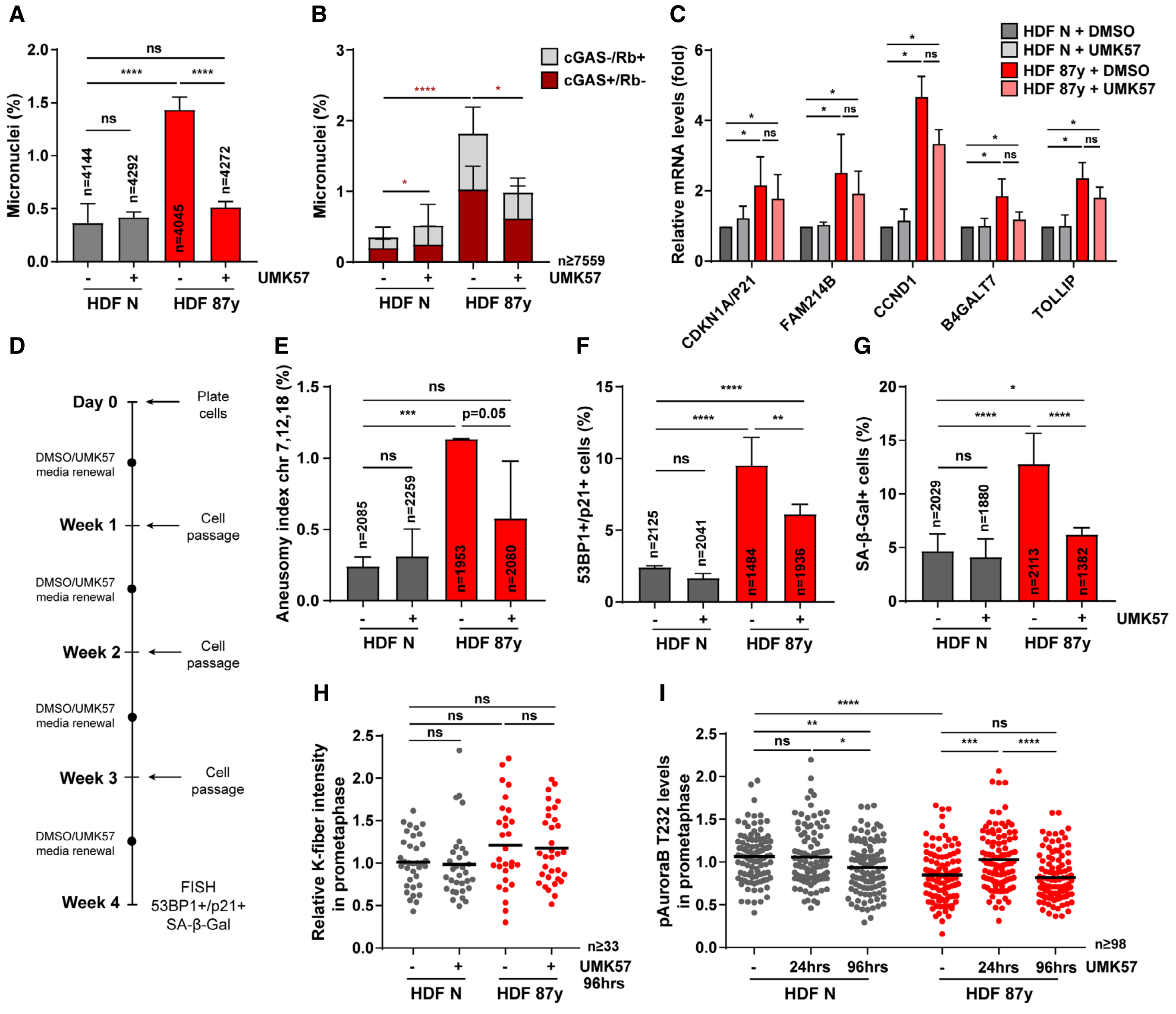

Figure EV5. Prolonged treatment with UMK57 inhibits CIN and senescence in cells from elderly donors (related to Fig 5).

A Percentage of micronuclei in n = cells of neonatal versus elderly samples after 96-h treatment.

B Percentage of cGAS^{+}/Rb^{-} and cGAS^{-}/Rb^{+} micronuclei in n = cells scored by immunofluorescence analysis upon 96-h treatment.

C Relative *CDKN1A/P21, FAM214B, CCND1, B4GALT7*, and *TOLLIP* transcript levels in total RNA of neonatal (HDF N; n = 5 replicates) and elderly (HDF 87 years; n = 3 replicates) fibroblasts treated with DMSO or UMK for 96 h. *TBP* and *HPRT1* were used as reference genes. All levels were normalized to DMSO-treated neonatal sample.

D Experimental layout for prolonged exposure to UMK57 of neonatal (N) and elderly (87 years) fibroblast cultures, with cell passage every week and media renewal halfway each week. At week 4, chromosome segregation and senescence biomarkers were analyzed.

E Aneusomy index of chromosomes 7, 12, and 18 measured by interphase FISH in n = cells.

F Percentage of n = cells staining positive for double immunostaining of Cdkn1a/p21 (cell cycle inhibitor) and 53BP1 (≥ 1 foci; DNA damage) senescence biomarkers.

G Percentage of n = cells staining positive for SA-β-gal activity.

H Calcium-stable k-fiber intensity levels scored by immunofluorescence analysis of $n \geq 33$ tubulin-stained mitotic cells in prometaphase of neonatal and elderly samples treated with DMSO (−) and UMK57 (+) for 96 h. Levels were normalized to neonatal DMSO-treated condition.

I Phospho-Aurora B Thr232 (pAuroraB T232) levels at kinetochores/centromeres of neonatal and elderly prometaphase cells treated with DMSO (−) or UMK57 (+) for 24 or 96 h. Intensity levels were normalized to ACA.

Data Information: All values are mean ± SD of at least two independent experiments. ns $P > 0.05$, *$P < 0.05$, **$P < 0.01$, ***$P < 0.001$, ****$P < 0.0001$ by two-tailed (A, B, E–G) chi-square and (C, H, I) Mann–Whitney tests.

© 2020 The Authors
