# Supplementary material for: Small‐molecule inhibition of aging‐associated chromosomal instability delays cellular senescence
Source: EMBO Rep. 2020 Mar 5;21(5):e49248. doi: 10.15252/embr.201949248 (PMC7202060; doi:10.15252/embr.201949248)
Supplement: Supplementary file 7 — Source Data for Expanded View [file EMBR-21-e49248-s009.zip › 49248_Source_Data_for_EV_Figures/Source_Data_for_FigEV2.pdf]

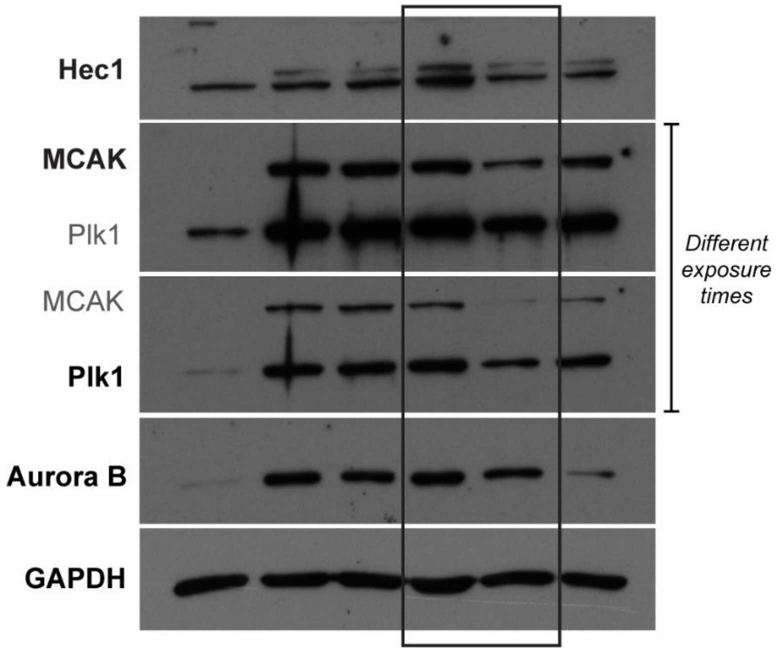

Source Data 3. Uncropped and unprocessed images of the Western blot results shown in Fig EV2B. Different exposure times of the same blot are shown for cases in which different proteins detected within the same blot were quantified using distinct exposures. The exposures that were considered for quantification are highlighted in bold black.
