# Supplementary material for: Small‐molecule inhibition of aging‐associated chromosomal instability delays cellular senescence
Source: EMBO Rep. 2020 Mar 5;21(5):e49248. doi: 10.15252/embr.201949248 (PMC7202060; doi:10.15252/embr.201949248)
Supplement: Supplementary file 7 — Source Data for Expanded View [file EMBR-21-e49248-s009.zip › 49248_Source_Data_for_EV_Figures/Source_Data_for_FigEV4.pdf]

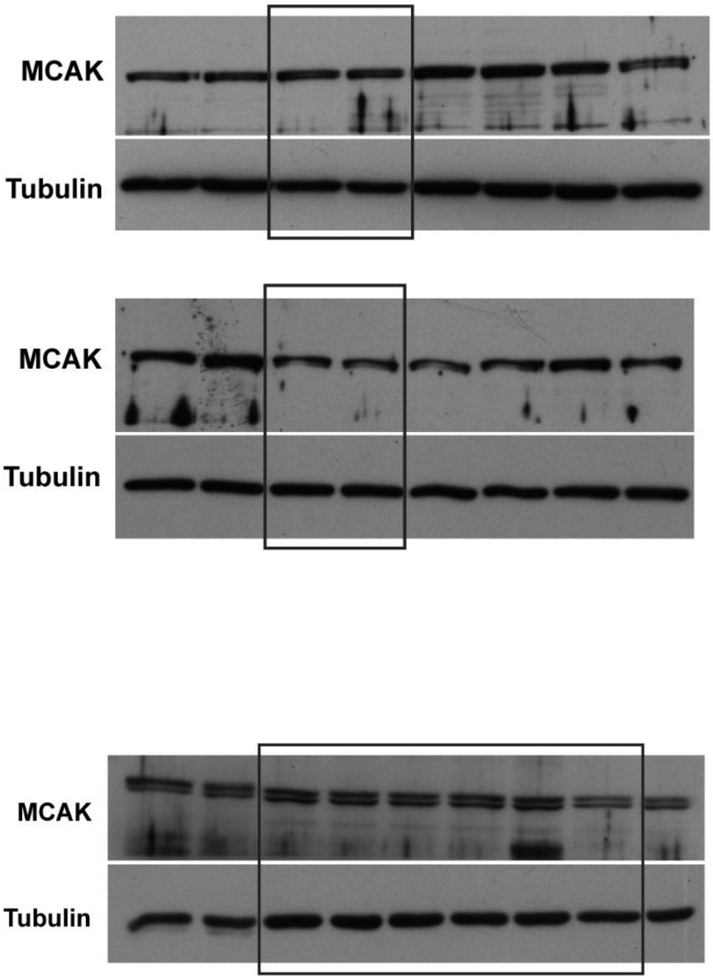

Source Data 5. Uncropped and unprocessed images of the Western blot results shown in Fig EV4E and EV4F.
