# Supplementary figures and images for: Small‐molecule inhibition of aging‐associated chromosomal instability delays cellular senescence
Source: EMBO Rep. 2020 Mar 5;21(5):e49248. doi: 10.15252/embr.201949248 (PMC7202060; doi:10.15252/embr.201949248)

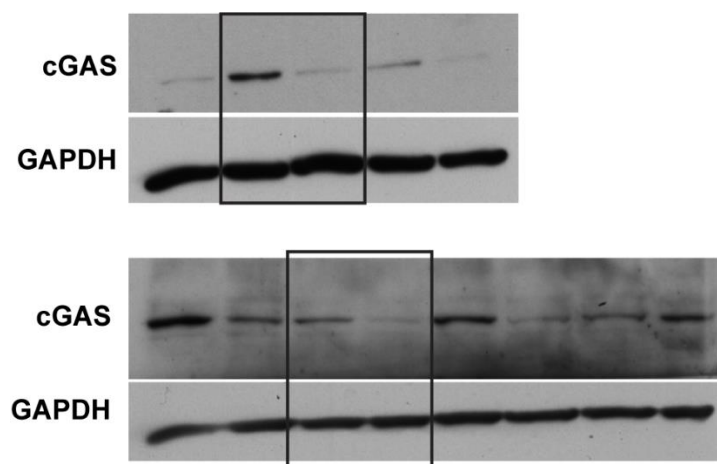

**Source Data 4.** Uncropped and unprocessed images of the Western blot results shown in Fig EV3E.

Supplement: Supplementary file 7 — Source Data for Expanded View [file EMBR-21-e49248-s009.zip › 49248_Source_Data_for_EV_Figures/Source_Data_for_FigEV3.pdf]

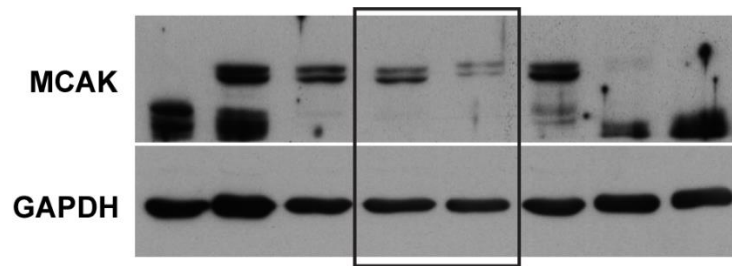

**Source Data 1.** Uncropped and unprocessed images of the Western blot results shown in Fig 1I.

Supplement: Supplementary file 9 — Source Data for Figure 1 [file EMBR-21-e49248-s007.pdf]

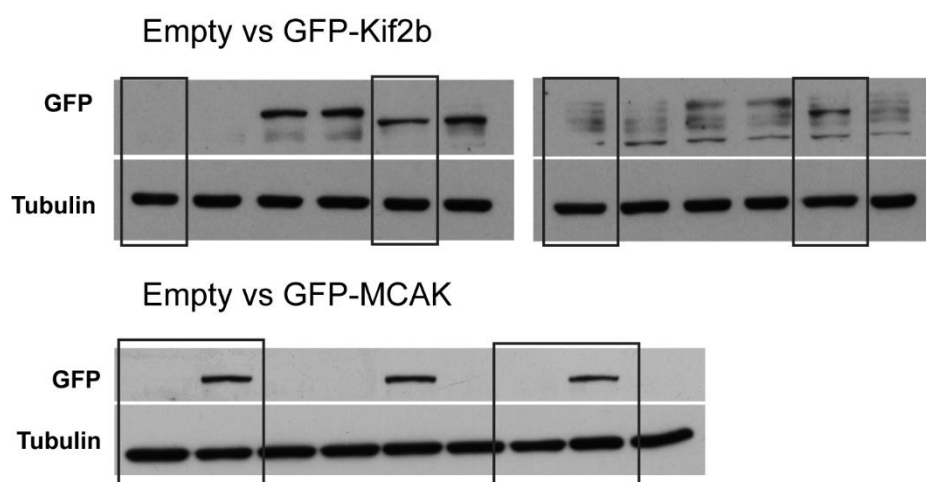

**Source Data 2.** Uncropped and unprocessed images of the Western blot results shown in Fig 2A.

Supplement: Supplementary file 10 — Source Data for Figure 2 [file EMBR-21-e49248-s008.pdf]
